# LINC00857 promotes colorectal cancer progression by sponging miR-150-5p and upregulating HMGB3 (high mobility group box 3) expression

**DOI:** 10.1080/21655979.2021.2003941

**Published:** 2021-12-07

**Authors:** Dongbing Zhou, Sijia He, Daquan Zhang, Zhenbing Lv, Jing Yu, Quanlin Li, Min Li, Wei Guo, Feng Qi

**Affiliations:** a Department of General Surgery, Tianjin Medical University General Hospital, Tianjin, China; b Department of Gastrointestinal Surgery, Nanchong Central Hospital, the Second Clinical Institute of North Sichuan Medical College, Nanchong, Sichuan, China; c Department of Medical Imaging, Nanchong Central Hospital, the Second Clinical Institute of North Sichuan Medical College, Nanchong, Sichuan, China

**Keywords:** LINC00857, miR-150-5p, HMGB3, colorectal cancer, TCGA

## Abstract

Colorectal cancer (CRC) is the third most commonly diagnosed malignant tumor worldwide. LINC00857 has been reported as a dysregulated long non-coding RNAs (lncRNAs) involved in the genesis and development of different cancers. In CRC, accumulating evidence indicates that high mobility group box 3 (HMGB3) is over-expressed and contributes to CRC development. However, the mechanism underlying HMGB3 upregulation in CRC remains unclear. The present work aims to investigate the role of LINC00857 and its functional interaction with HMGB3 in regulating CRC progression. Differential expression of LINC00857 between CRC tissues and normal tissues was identified in TCGA (The Cancer Genome Atlas) database. *In vitro* functional assays were performed to explore the biological functions of LINC00857 in CRC cells. *In vivo* xenograft model was employed to investigate the role of LINC00857 in CRC tumorigenesis. We found that LINC00857 was significant upregulated in CRC tissues and cell lines. LINC00857 knockdown significantly inhibited the proliferation, migration and invasion of CRC cells, and also induced apoptosis. Moreover, LINC00857 knockdown suppressed CRC tumorigenesis *in vivo*. We further demonstrated that the effects of LINC00857 in CRC cells were mediated through miR-150-5p/HMGB3 axis. LINC00857 negatively regulates the activity of miR-150-5p, which releases its inhibition on HMGB3 expression. Our data indicate that LINC00857/miR-150-5p/HMGB3 axis plays a fundamental role in regulating the malignant phenotype and tumorigenesis of CRC. Targeting this axis may serve as novel therapeutic strategies for CRC treatment.

## Introduction

Although considerable advances have been achieved in cancer treatment, colorectal cancer (CRC) remains as a challenge and is ranked as the second common causes of cancer-related death worldwide [1]. Surgical resection followed by radio- and chemotherapy is the mainstay for CRC treatment [2]. However, the 5-year survival rate is still poor due to metastasis and local recurrence [3]. Emerging evidences revealed that CRC carcinogenesis is associated with multiple genomic variations [4]. The recent application of single-cell RNA-sequencing (scRNA-seq) offers unprecedented opportunities to understand the genetic alterations related to CRC [5].

Long noncoding RNAs (lncRNAs) are non-coding transcripts that are over 200 nucleotides in length [6,7]. Accumulating evidence shows that lncRNAs act as oncogenes or tumor suppressor genes in the initiation or progression of different cancers [8–10]. LncRNAs can regulate gene expression via multiple mechanisms, such as sponging microRNAs (miRNAs) [11]. Recently, multiple lncRNAs were reported to play important roles in the genesis, metastasis and invasion of CRC [12–14]. For instance, lncRNA GLCC1 contributes to CRC carcinogenesis through stabilizing c-Myc and regulating glycolytic metabolism [15]. LncRNA MALAT1 promotes CRC development by activating PRKA (kinase anchor protein 9) [16]. Moreover, lncRNA PCAT-1 modulates CRC metastasis by promoting PRC2 expression [17]. Recently, lncRNA LINC00857 is found to be overexpressed in hepatocellular carcinoma (HCC), and it promotes the progression of lung cancer [18,19]. However, the potential biological functions of LINC00857 in CRC is unknown.

High mobility group box 3 (HMGB3) belongs to the high-mobility group box (HMGB) family comprising HMGB1, HMGB2, HMGB3 and HMGB4 [20]. The HMGB members are chromatin-remodeling proteins involved in DNA replication, repair, recombination and transcription [21–24]. Previous studies showed that HMGB3 is implicated in the development of esophageal squamous cell cancer (ESCC), leukemia, gastric cancer (GC), urinary bladder cancer and non-small cell lung cancer (NSCLC) [25–29]. Overexpression of HMGB3 has also been reported in CRC patients, which enhances cell proliferation and migration [30]. However, the regulatory mechanism of HMGB3 upregulation in CRC is remains unclear.

The present study aims to determine the expression pattern of LINC00857 in colon adenocarcinoma (COAD) patients and CRC cell lines. We also attempt to investigate the molecular mechanisms underlying the functional role of LINC00857 in CRC. Based on the data in TCGA (The Cancer Genome Atlas) database, we firstly identified that LINC00857 was significantly upregulated in COAD patients. Its upregulation was further confirmed in CRC samples collected in this study, which was correlated with a dismal prognostic outcome of the patients. As revealed by functional experiments, LINC00857 served as an oncogenic factor to promote the proliferation, invasion and migration of CRC cells. Our data also revealed that LINC00857 interacted with miR-150-5p to negatively regulate its activity, which released the inhibition of miR-150-5p on HMGB3 expression in CRC cells. Collectively, our study delineated the functional roles of LINC00857/miR-150-5p/HMGB3 axis in regulating the progression of CRC.

## Materials and methods

### Patients and specimen collections

Tumor samples and adjacent non-carcinoma tissue samples were collected from 50 CRC patients by surgical resection at Nanchong Central Hospital. The adjacent normal tissues were obtained from the resected colon tissues (≥10 cm away from tumor position). After collection, samples were snap-frozen in liquid nitrogen and preserved at − 80ʹ before RNA extraction. Sample collection and experimental procedures (from January of 2018 to August of 2020) were approved by the ethics Committee of Nanchong Central Hospital, The Second Clinical Institute of North Sichuan Medical College (No.20200287). All patients agreed with the usage of samples and clinical data in scientific research, and informed consent was obtained from all patients.

### Cell culture and transfection

The human CRC cell lines (HCT116, HCT8, HT29, SW620, SW480, LOVO), and normal colonic epithelial cells (FHC) were purchased from the Cell Bank of the Chinese Academy of Sciences (Shanghai, China). All the cells were cultured in DMEM (Gibco, USA) containing 10% fetal bovine serum (FBS, Gibco, USA), 0.1% penicillin-streptomycin (Gibco, USA), in a humidified incubator under 37 ʹ and 5% CO_2_.

miR-NC (UCACAACCUCCUAGAAAGAGUAG), miR-150-5p mimic (UGGCAGUGUCUUAGCUGGUUGU) and miR-150-5p inhibitor (UCUCCCAACCCUUGUACCAGUG) were purchased from Guangzhou RiboBio (Guangzhou, China). SW480 and HCT116 were transfected with 2 µM miR-NC, 2 µM miR-150-5p mimic and 2 µM miR-150-5p inhibitor using Lipofectamine 2000 (Invitrogen, CA, USA) according to the manufacturer’s instructions. Briefly, cells were seeded in 6-well plates at a density of 5 × 10^5^ cells/well. 24 hours later, 2 µM of each molecule was added into 100 µl Opti-MEM® I Reduced-Serum Medium (Invitrogen, CA, USA), and then 6 µL Lipofectamine 2000 reagent was added for 10 min incubation at room temperature. The mixture was added to cell culture dropwise, and the transfected cells were subjected to subsequent analysis 48 hours post-transfection.

### Dual luciferase reporter assay

DNA fragments carrying wild type or mutant LINC00857/HMGB3 3′UTR were cloned into psiCHECK-2 reporter vector. The reporter plasmids were co-transfected with miR-150-5p mimic or miR-NC into SW480 or HCT116 cells using Lipofectamine 2000. After 48 hours, luciferase activity was measured using Dual-Luciferase Reporter 1000 System with Dual-Glo Luciferase Assay kit (E2920; Promega Corporation). The luciferase activity was measured on a Synergy H1 microplate reader using luminescence measurement mode (Winooski, Vermont, USA). The firefly luciferase activity in the reporter plasmid was normalized to that of Renilla luciferase activity.

### Stable LINC00857 knockdown by lentiviral transduction

LINC00857 sequence was retrieved from NCBI databank (https://www.ncbi.nlm.nih.gov/). Sh-NC (F: 5ʹUGUAAGUACGGUGGAGAAUU3ʹ, R:5ʹGCCCUCAUCUUAACCUAACG3ʹ); sh-LINC00857 sequence (F: 5ʹGGUAAGGGAAGGUGGAGAAUU3ʹ, R:5ʹUUCUCCACCUUCCCUUACCUU3ʹ) was constructed into PLKO-Puro lentiviral vector (Sigma, St Louis, MO, USA). For lentiviral production, pMD2.G (2 µg) and psPAX2 (1 µg) lentivirus package vectors (Addgene, USA) were co-transfected with sh-LINC00857 plasmid (1 µg) or empty PLKO-Puro vector (1 µg) into the human embryonic kidney cells (HEK293T, ATCC) using Lipofectamine 2000. After 48 hours, the supernatant of cell culture containing lentiviral particles was centrifuged at 2000 g for 5 minutes, respectively. The supernatant was collected and snap-frozen by liquid nitrogen, and stored at −80ʹ until further usage.

To generate stable shRNA-mediated knockdown, 1 × 10^5^ SW480 or HCT116 cells were seeded in a 24-well plate. When cells reached at 50 ~ 60% confluence, cells were infected with recombinant lentivirus (sh-LINC00857 or sh-NC) at a MOI (multiplicity of infection) = 5, in the presence of 10 µg polybrene (Sigma, tr-1003-g). Infected cells were selected with 1.0 μg/mL puromycin for two weeks to eliminate the uninfected cells before further experiment.

### RNA isolation and quantification

TRIzol reagent (Invitrogen, Carlsbad, CA, USA) was utilized to isolate total RNA from tissues or cell lines according to the instructions. For nucleoplasm fraction experiment, the nuclear and cytoplasmic faction was extracted using NE-PER™ Nuclear and Cytoplasmic Extraction Reagents (Thermo Fisher Scientific, CA, USA, Cat# 78833). The extracted total RNA was dissolved in DEPC water and its concentration and quality were determined with NanoDrop spectrometer (A260/280 is around 1.8). cDNA was synthesized from 1 µg total RNA by reverse transcription with the PrimeScript RT reagent kit (RR036A, Takara Biotechnology, Shiga, Japan). Real-time qPCR was performed using SYBR® Premix Ex TaqTM II kit (RR820A, Takara Biotechnology, Shiga, Japan) on the ABI7500 Real-Time fluorescence quantitative PCR System (Applied Biosystems, Carlsbad, CA, USA). The PCR cycling condition used: 95ʹ 5 mins, 40 cycles of 95ʹ 30 sec, 60ʹ 30 sec and 72ʹ 60 sec. 2–∆∆Ct method was used to analyze the relative expression level and Glyceraldehyde-3-phosphate dehydrogenase (GAPDH) was used as the internal reference gene. All primers were synthesized by Takara Biotechnology (Dalian, China). The primer sequences were as follows:

LINC00857 Forward: CCCCTGCTTCATTGTTTCCC,

LINC00857 Revise: AGCTTGTCCTTCTTGGGTACT,

miR-150-5p Forward: TCGGCGTCTCCCAACCCTTGTAC,

miR-150-5p Reverse: GTCGTATCCAGTGCAGGGTCCGAGGT,

HMGB3 Forward: ATTCGGAATTCCGTATCTGGCCTTTTGAC,

HMGB3 Reverse: CGGTTACTCGGCTTACGCTTGGACTG,

U6 Forward: GCTTCGGCAGCACATATACTAAAAT,

U6 Reverse: CGCTTCACGAATTTGCGTGTCAT,

GAPDH Forward: GGAGCGAGATCCCTCCAAAAT,

GAPDH Reverse: GGCTGTTGTCATACTTCTCATGG.

### Cell proliferation assay

CCK-8 (Key GEN Bio TECH, China) assay was conducted to evaluate cell proliferation in accordance with manufacturer’s protocols. About 1 × 10^3^ SW480 or HCT116 cells after LINC00857 knockdown were seeded into a 96-well plate. Cells were incubated for 24, 48 and 72 hours. At each time point, CCK-8 solution (10 µl) were added to each well, and the cells were incubated for 2 hours in the cell culture incubator. Absorbance (OD) value at 450 nm was detected with a microplate reader (Infinite M200 Pro, Tecan).

### Colony formation experiment

2.5 × 10^3^ cells were plated into a six-well plate, and cultured for 2 weeks in the presence of indicated treatments. The culture medium was changed every 3 days during the period. After 14 days, cells were fixed with 100% Methanol for 15 min and fixed cells were stained with 0.1% crystal violet staining solution (Beyotime, China) for 15 mins. Subsequently, the number of colonies was counted and the morphology of the colonies was photographed under Leica AM6000 microscope (Leica, Wetzlar, Germany).

### Transwell migration and invasion

The migration and invasion abilities of cells after indicated treatments were determined using BD BioCoat™ BD Matrigel™ Invasion Chamber (BD Biosciences, Franklin Lakes, NJ, USA) in accordance with the manufacturer’s instructions. The transwell upper chamber without Matrigel (BD Biosciences, 356234) was used for migration assay, while transwell upper chamber coated with Matrigel was used for invasion assay. 6 × 10^5^ Cells were inoculated into transwell upper chamber in serum-free medium and 1000 μL of 10% serum-containing medium was added to the lower chamber. After 24 hours, cells on the transwell membrane were fixed with 4% paraformaldehyde at room temperature for 10 mins and stained with 0.5% crystal violet (Beyotime, Shanghai, China)) for 15 mins. Cells were photographed under Leica AM6000 microscope (Leica, Wetzlar, Germany) at 400x magnification. The cell image was analyzed with Image J software (Bethesda, MD, USA).

### TCGA analysis

Data consisting of 471 COAD patient samples and 41 normal tissues were retrieved from the TCGA database (https://cancergenome.nih.gov/). Gene expression profiles from TCGA database were utilized to compare LINC00857 expression level between COAD samples and normal tissues using the online tool.

### Target prediction

LncBASE database was used to predict the target miRNAs which potentially interact with LINC00857 as described in the previous study [31]; The starBase v2.0 was used to predict the regulatory target of miR-150-5p as described in the previous study [32].

### RNA pull-down assays

Biotin-labeled miR-150-5p (WT: UGGCAGUGUCUUAGCUGGUUGU) or miR-NC (UCACAACCUCCUAGAAAGAGUAG) were synthesized by Ruibiotech (Beijing, China). 5 × 10^6^ cells were lysed in 1000 µL RIPA buffer and 10 µM biotinylated oligonucleotide was added to 500 µL lysate sample for 1-hour incubation. The remaining 500 µL lysate was used as the input. Then the lysate with biotinylated oligonucleotide was mixed with 100 µL Pierce™ Streptavidin magnetic beads (Thermo Fisher Scientific, 88816) for 2-hour incubation. The bead-bound samples were separated using a magnetic stand, and the beads were washed for 4 times with 1000 µl RIPA buffer. Total RNA was extracted from the beads or the input samples using TRIzol Reagent. The cDNA synthesis and qPCR procedures were described in RNA isolation and quantification section. The relative amount of precipitated LINC00857 in each sample was normalized to the input.

### Apoptosis assay

FITC Annexin V Apoptosis Assay Kit (BD Biosciences) was used for cell apoptosis assay. Cells with different treatments were trypsinized and washed twice with 1xPBS, and resuspended in the staining solution. In brief, 5 μL Annexin V-FITC and 5 μL propidium iodide (PI) were added to the 1000 μL cell resuspension with 1 million cells, and incubated for 30 mins in the dark. Stained cells were centrifuged and washed twice with staining buffer, and resuspended in 400 μL buffer. The percentage of apoptotic cells was detected by BD FACS CantoTM II Flow Cytometer (BD Biosciences). Single positive staining samples were used for compensation of fluorescence signal spillover between two channels. The analysis was performed using FlowJo™ v10.8 (FlowJo.LLC).

### Western blotting (WB) assay

Total protein was extracted from cells using RIPA lysis buffer containing protease inhibitor cocktail (Thermo Fisher Scientific, Waltham, MA, USA, Cat# 89900). Cells suspended in RIPA buffer were lysed on ice for 10 mins and lysed cells were centrifuged at 14000 rpm for 10 mins. The supernatant containing total protein lysate was quantified by a BCA Protein assay kit (Beyotime; Shanghai, China, Cat# 5000 T). 20 µg protein was used for SDS-PAGE electrophoresis in 8% SDS-PAGE gel, followed by transfer onto the nitrocellulose membranes. Afterward, the membranes were blocked by 5% milk for 1 hour, followed by incubation with anti-HMGB3 antibody (R&D Systems, Minneapolis, MN, USA, Cat# MAB5507, 1:1000 dilution) and anti-GAPDH antibody (Santa Cruz Biotechnology, TX, USA, Cat# sc-365062, 1:1000 dilution) overnight at 4ʹ. After 4 washes with TBST buffer, the membranes were incubated with HRP-conjugated secondary antibody (Cell Signaling Technologies, MA, USA, Cat# 7074, 1:3000 dilution) at room temperature for 1 hour. Enhanced chemiluminescence kit (Santa Cruz, TX, USA, Cat# sc-2048) was used for protein band development and the membranes were photographed on the GelDoc Go Gel Imaging System (Bio-Rad, CA, USA). The densitometry analysis was performed with Image J software (Bethesda, MD, USA).

### In vivo xenograft tumorigenesis

The Research Ethics Committee of Nanchong Central Hospital approved the animal experiment procedures (Approval number: 20170287). A total of 12 BALB/c nude mice (male, 4–6 weeks old, weighing 16–20 g) were provided by the College of Veterinary Medicine of Yang Zhou University. Mice were maintained in environment of the temperature at 22 ± 1°C, relative humidity of 55 ± 1% and with a 12 h light/dark cycle. 1 × 10^6^ HCT116 cells transduced with sh-LINC00857 or sh-NC lentivirus after puromycin selection were suspended in 200 uL PBS/Matrigel mixture (1:1 in volume), and were injected subcutaneously into two front flanks of 1 × 10^6^ of the nude mice (n = 6 in each group). The length (a) and width (b) of xenograft tumor were measured by the Vernier caliper, and the volume was determined by the formula: tumor volume = 1/2 × a× b^2^. Tumor volume was recorded every 7 days. After 35^th^ d, all mice were anesthetized with 150 mg/kg pentobarbital sodium and sacrificed by cervical dislocation. The tumors were collected by surgical removal and weighed.

### Statistical analysis

Data were analyzed by GraphPad Prism software (version, 7.04). Quantitative data were expressed in mean ± SD from 3 independent experiments. Two-tailed Student’s t-tests were conducted to analyze the statistical difference between two groups. Comparisons among multiple groups were analyzed using one-way analysis of variance (ANOVA) with Tukey’s post hoc test for pairwise comparison. Comparisons of data at multiple time points were examined using two-way ANOVA. Kaplan Meier Curve and log-rank test were used to compare the cumulative survival rates in CRC patients. Spearman correlation analysis was performed to determine the correlation between two genes. P < 0.05 was considered to be statistically different. p < 0.05: *, p < 0.05; **, p < 0.01; ***.

## Results

This study aims to determine the expression pattern of LINC00857 and its functional role in CRC. We also investigated the molecular mechanisms underlying the functional role of LINC00857 in CRC. Based on the data in TCGA database, we firstly identified that LINC00857 was significantly upregulated in colorectal cancer patients. Its upregulation was confirmed in CRC samples collected in this study, which was correlated with a dismal prognostic outcome of the patients. As revealed by functional experiments, LINC00857 served as an oncogenic factor to promote the proliferation, invasion and migration of CRC cells. LINC00857 overexpression is required for CRC cell proliferation, migration and invasion. LINC00857 upregulation maintains HMGB3 protein expression by sponging miR-150-5p. In addition, LINC00857 knockdown remarkably suppresses CRC tumorigenesis in mouse xenograft model.

### LINC00857 is significantly upregulated in CRC tissues and cell lines

**Figure 1.**
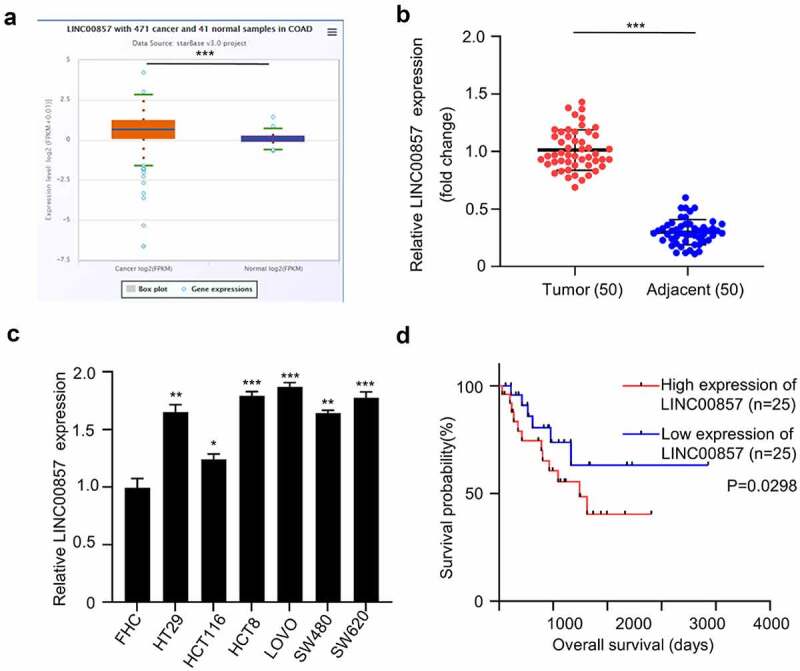
Increased expression LINC00857 were identified in colon cancer tissue and cells.(a) LINC00857 expression in colon adenocarcinoma (COAD) (croci box) and normal tissue (blue box) from TCGA databse (P = 0.00052). (b) qPCR analysis of LINC00857 expression in CRC tumors and adjacent normal tissues collect in this study (n = 50 pairs). (c) qPCR analysis of LINC00857 in the CRC cell lines (HT29, HCT116, HCT8, LOVO, SW620 and SW480) and normal human colonic epithelial cell line FHC. (d) Kaplan Meier Curve and log-rank test showed that high expression of LINC00857 was correlated with a poor prognosis in CRC patients. *P < 0.05, **P < 0.01, ***P < 0.001

To determine LINC00857 expression profile in colon cancer, TCGA data consisting of 471 COAD patients and 41 normal colon tissues were analyzed. We found that LINC00857 expression level was significantly increased in COAD samples as compared with normal samples (Figure 1(a)). In order to confirm the upregulation of LINC00857 in CRC, we collected 50 tumor tissues and adjacent non-carcinoma samples from 50 CRC patients. qRT-PCR analysis revealed the significant upregulation of LINC00857 in CRC tumor samples (Figure 1(b)). Furthermore, we analyzed the expression of LINC00857 between colorectal cancer (CRC) cell lines (HT29, HCT116, HCT8, LOVO, SW620 and SW480) and normal human colonic epithelial cell line FHC. qRT-PCR analysis showed a significant increase of LINC00857 level in all CRC cell lines as compared to the normal colonic epithelial cells (Figure 1(c)). SW480 and HCT116 256 cell line with relatively lower LINC00857 expression were chosen for further knockdown experiment.

We next sought to examine whether LINC00857 expression level correlates with patient survival. The median value of LINC00857 expression was used as the cutoff to divide the patients into high (n = 25) and low (n = 25) expression group. Kaplan Meier Curve and log-rank test showed that high expression of LINC00857 was significantly correlated with a poor prognosis in CRC patients (Figure 1(d)). In addition, high expression of LINC00857 was also significantly associated with malignant features such as poor differentiation and lymph node metastasis (Table 1). Collectively, the above data show that LINC00857 is significantly upregulated in CRC, and is correlated with dismal prognostic outcome of CRC patients.

**Table 1.** LINC00857 expression and clinical characteristics of the CRC patients

	LINC00857 expression			
Clinicopathological parameters	Low (n = 25)	High (n = 25)	*P* value	Patients Characteristic(n = 50)
Age			0.208	*Age, median (interquartile range), y*
≤60	20	16		*51.36 (32–75)*
>60	5	9		
Gender			0.239	*No. (%)*
Male	14	18		*32 (64)*
Female	11	7		*18(36)*
Tumor size			0.087	*Tumor size, median (interquartile range), cm*
≤2 cm	17	11		*2.47 (1.26–6.85)*
>2 cm	8	14		
TNM stage			0.208	TNM stage, No. (%)
I/II	20	16		36 (72)
III/IV	5	9		14 (28)
Tumor differentiation			0.015	Tumor differentiation, No. (%)
High-moderate	12	4		16 (32)
Low	13	21		34 (68)
Vascular invasion			0.047	Vascular invasion, No. (%)
Yes	3	9		12 (24)
No	22	16		38 (76)
Distant metastasis			0.066	Distant metastasis, No. (%)
Positive	2	7		9 (18)
Negative	23	18		41 (82)
Lymph node metastasis			0.021	Lymph node metastasis, No. (%)
Positive	1	7		8 (16)
Negative	24	18		42 (84)

### LINC00857 knockdown inhibits cell proliferation, and induces apoptosis in CRC cells

**Figure 2.**
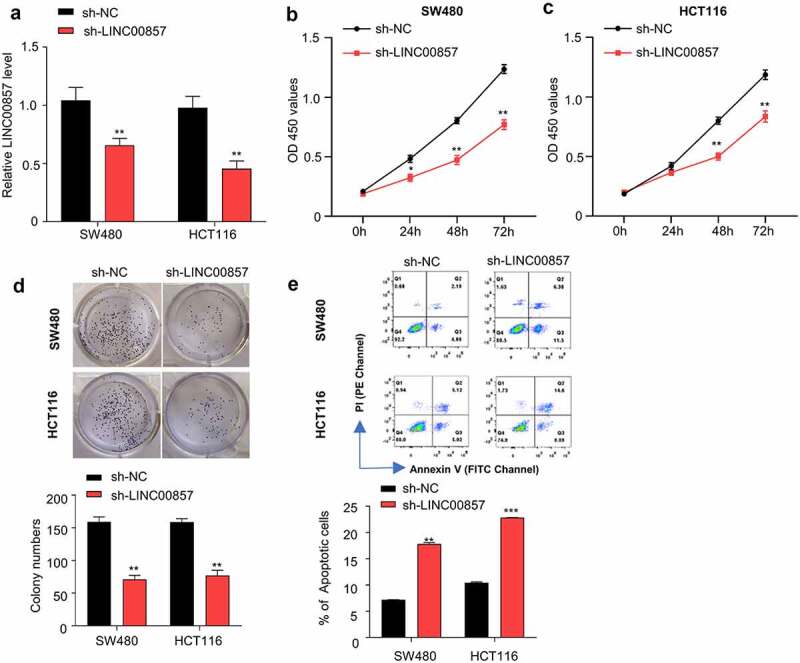
LINC00857 silencing inhibits CRC cell proliferation and promotes their apoptosis. HCT116 and SW480 cells were stably transduced with lentivirus expressing sh-NC or sh-LINC00857. (a) LINC00857 expression level was measured by qRT-PCR. (b) SW480 and (c) HCT116 cells expressing sh-NC or sh-LINC00857 were subject to CCK-8 proliferation assay. (d) HCT116 cells expressing sh-NC or sh-LINC00857 were subject to colony formation assay. (e) HCT116 cells expressing sh-NC or sh-LINC00857 were subject to Annexin V/PI staining for apoptosis analysis. Cells in quadrat 2 and 3 were considered as apoptotic cells. *P < 0.05, **P < 0.01, ***P < 0.001

We selected SW480 and HCT116 cell lines in which LINC00857 were upregulated by different extent for functional study (Figure 1(c)). HCT116 and SW480 cells were transduced with lentivirus carrying sh-LINC00857 or sh-NC, and then selected by puromycin for two weeks to enrich the transduced cells. qRT-PCR analysis revealed that sh-LINC00857 was able to efficiently reduce LINC00857 expression level in both HCT116 and SW480 cells (Figure 2(a)). CCK-8 proliferation assay demonstrated that LINC00857 knockdown significantly suppressed the proliferation of SW480 and HCT116 cells (Figure 2(b,c)). Consistently, LINC00857 knockdown impaired the colony formation capacity of SW480 and HCT116 cells (Figure 2(d)). Furthermore, Annexin V/PI apoptosis assay revealed that LINC00857 silencing significantly promoted the percentage of apoptotic events in SW480 and HCT116 cells (Figure 2(e)). Collectively, these findings indicate that LINC00857 is indispensable to support the proliferation and survival of SW480 and HCT116 cells *in vitro*.

### LINC00857 knockdown impairs the migration and invasion of CRC cells

**Figure 3.**
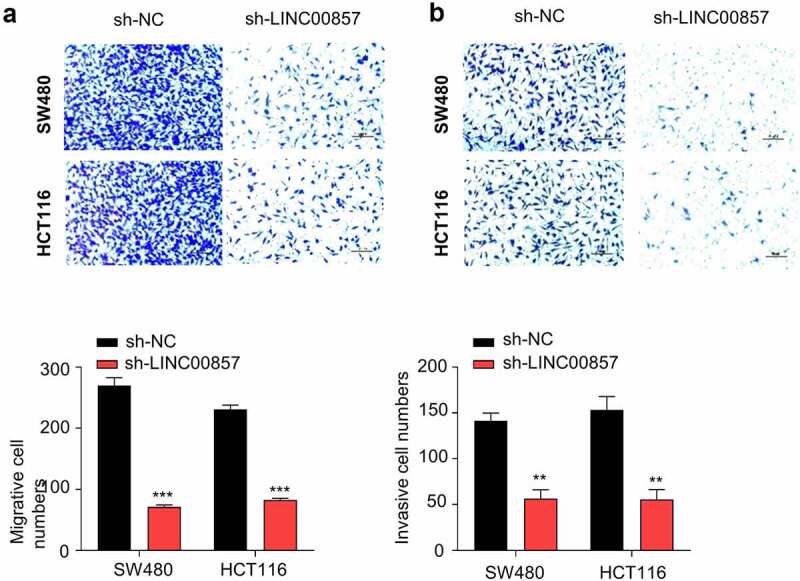
LINC00857 knockdown suppresses CRC cell migration and invasion. HCT116 and SW480 cells were stably transduced with lentivirus expressing sh-NC or sh-LINC00857. (a) Transwell migration and (b) invasion assays were performed in sh-NC and sh-LINC00857 cells. Representative images of assay results (upper panel), statistic summary of cell number counts (lower panel). Scale Bar: 50 µM. **P < 0.01, ***P < 0.001

To further investigate whether LINC00857 supports the malignant phenotype of CRC cell, we performed transwell migration and invasion assay in HCT116 and SW480 cells. LINC00857 knockdown significantly impaired the migration ability and the invasion capacity in both SW480 and HCT116 cells (Figure 3(a,b)). Together, the results suggest that LINC00857 is required to support the invasion and migration of CRC cells.

### LINC00857 acts as a sponge of miR-150-5p

**Figure 4.**
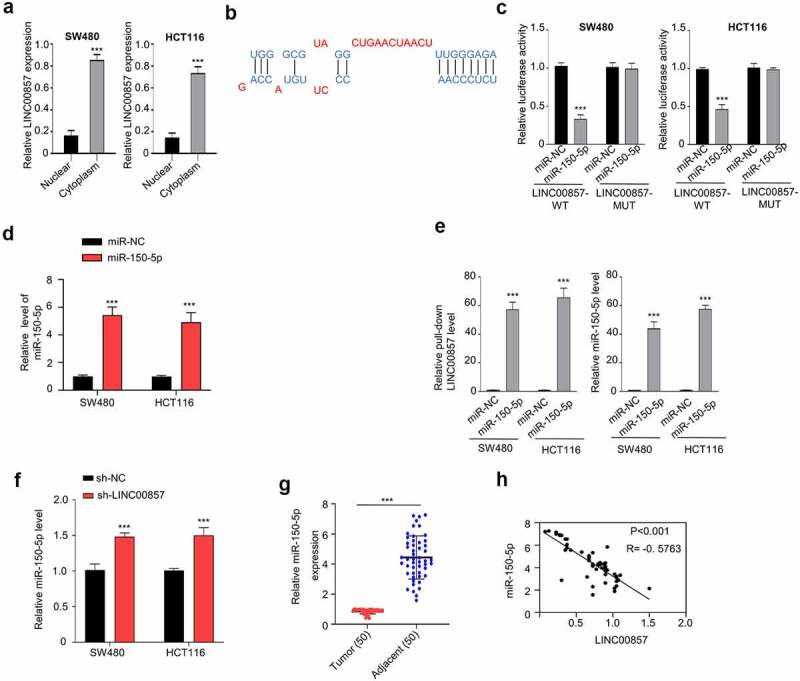
LINC00857 targets miR-150-5p within the CRC cells. (a) Cytoplasmic and nuclear separation of HCT116 and SW480 cells were collected and relative amount of LINC00857 in each fraction was determined by qRT-PCR. (b) Binding site for miR-150-5p in LINC00857 was predicted using lncBASE database. (c) WT or MUT luciferase reporter plasmids were transfected into SW480 and HCT116 cells for 48 h in the presence of miR-150-5p mimic or miR-NC. The luciferase activity was measured in each group. (d) miR-150-5p mimic and miR-NC was transfected into cells and the expression level of miR-150-5p was determined by qRT-PCR. (e) RNA-pull-down assay using Biotin-labeled miR-150-5p (WT) or miR-NC probes. Results indicated the enrichment of LINC00857 by miR-150-5p probe in CRC cells. (f) miR-150-5p level was upregulated when LINC00857 was knocked down in HCT116 and SW480 cells. (g) Relative miR-150-5p levels in CRC tumors and adjacent non-carcinoma tissues (n = 50 pairs). (H) LINC00857 expression level was negatively correlated with miR-150-5p expression level in CRC tumor samples. *P < 0.05, **P < 0.01, ***P < 0.001

We next sought to investigate the molecular mechanism underlying the oncogenic role of LINC00857. We first performed the nuclear and cytoplasmic separation and isolated RNA in both fractions. As revealed by qRT-PCR analysis, LINC00857 was mainly located in the cytoplasm (Figure 4(a)). To search for the potential downstream targets, we used the lncBASE database to find that LINC00857 has a binding site for miR-150-5p at its 3ʹUTR (Figure B). The wildtype (WT) and mutated (MUT) binding sites were cloned into luciferase reporter respectively. Dual-luciferase reporter assay showed that transfection of miR-150-5p mimic significantly inhibited LINC00857-WT reporter activity, but no inhibition was observed in LINC00857-MUT reporter (Figure 4(c)). We also confirmed that the transfection of miR-150-5p mimic enhanced the expression level of miR-150-5p mimic (Figure 4(d)). To validate the physical interaction between LINC00857 and miR-150-5p, we performed RNA-pull-down assay using Biotin-labeled miR-150-5p (WT) or miR-NC. Our results showed that miR-150-5p (WT) probe significantly enriched LINC00857 as compared to the miR-NC probe (Figure 4(e)). We further showed that miR-150-5p expression was significantly increased after LINC00857 knockdown (Figure 4(f)). We also detected miR-150-5p expression level in CRC tumors and para-cancerous normal tissues. qRT-PCR analysis revealed that miR-150-5p level was remarkably reduced in CRC tumor samples (Figure 4(g)). Spearman correlation coefficient analysis in 50 CRC tissues further revealed a negative correlation between the expression levels of LINC00857 and miR-150-5p (Figure 4(h)). Taken together, our data suggest that LINC01207 negatively regulates the expression of miR-150-5p in CRR cells.

### LINC00857 regulates the malignant features of CRC by sponging miR-150-5p

**Figure 5.**
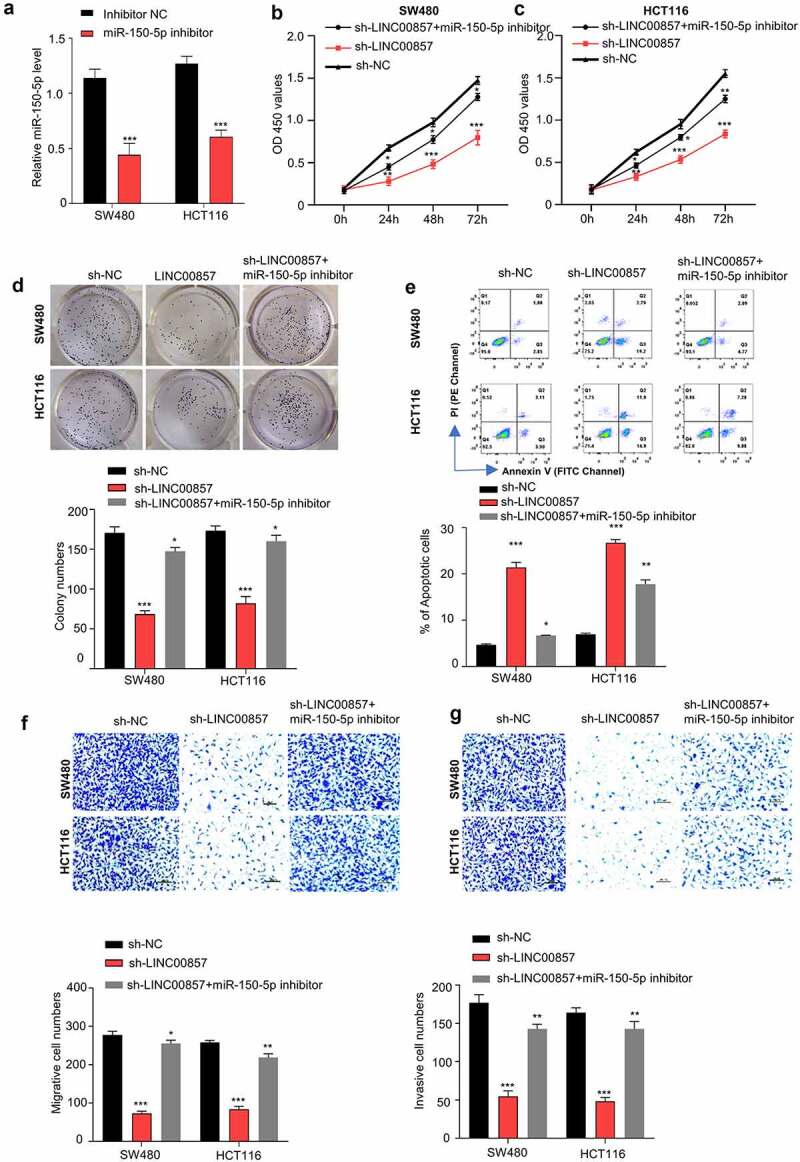
LINC00857 modulates CRC progression through sponing miR-150-5p. (a) miR-150-5p inhibitor suppressed miR-150-5p level as detected by qRT-PCR. (b) & (c) miR-150-5p inhibitor rescued the inhibition of LINC00857 silencing on proliferation of SW480 and HCT116 cells, as examined by CCK-8 assay. (d) miR-150-5p inhibitor rescued the inhibitory effect of LINC00857 silencing on colony formation of SW480 and HCT116 cells. (e) Annexin V/PI staining for apoptosis analysis. Cells in quadrat 2 and 3 were considered as apoptotic cells. (f & g) miR-150-5p inhibitor rescued the inhibitory effect of LINC00857 silencing on the migration (f) and invasion (g) of SW480 and HCT116 cells. Scale Bar: 50 µM. For all the above experiments: sh-NC: cell stabling expressing control shRNA; sh-LINC00857: cells stably expressing sh-LINC00857; sh-LINC00857+ miR-150-5p inhibitor: cells stably expressing sh-LINC00857 were transfected with miR-150-5p inhibitor. *P < 0.05, **P < 0.01, ***P < 0.001

To validate the involvement of miR-150-5p in the oncogenic role of LINC00857, we applied miR-150-5p inhibitor that could suppress the level of miR-150-5p (Figure 5(a)). We transfected miR-150-5p inhibitor to the cell with LINC00857 knockdown to investigate whether inhibiting miR-150-5p could rescue the effect of LINC00857 knockdown. CCK8 proliferation assay showed that knockdown of LINC00857 impaired the proliferation of SW480 and HCT116 cells, which was largely rescued by miR-150-5p inhibitor (Figure 5(b,c)). Consistently, the effect of LINC00857 knockdown on colony formation capacities was also significantly rescued by miR-150-5p inhibitor (Figure 5(d)). Moreover, the presence of miR-150-5p inhibitor significantly suppressed the apoptosis induced by the knockdown of LINC00857 (Figure 5(e)). Furthermore, the migration and invasion abilities of HCT116 and SW480 cells, which were suppressed after LINC00857 knockdown, were also largely increased in the presence of miR-150-5p inhibitor (Figure 5(f,g)). Taken together, our data suggest that miR-150-5p is a downstream effector mediating the oncogenic phenotype by LINC00857.

### LINC00857 enhances HMGB3 level by sponging miR-150-5p in CRC cells

**Figure 6.**
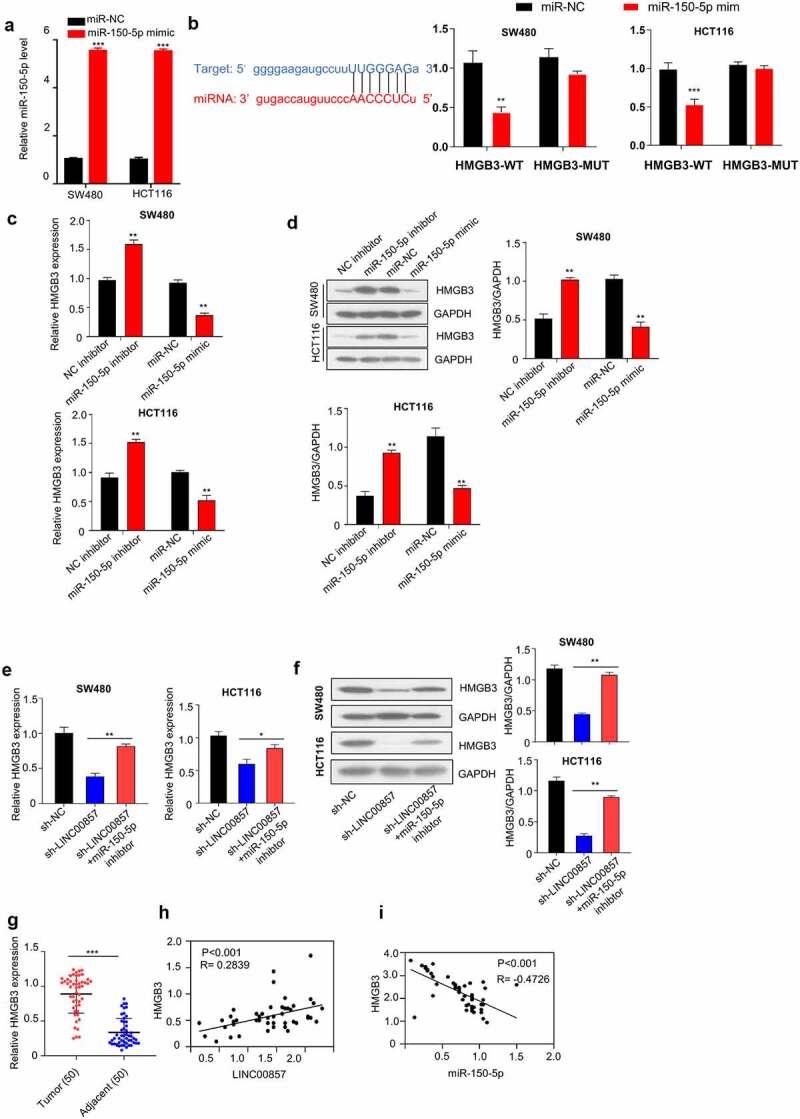
LINC00857 enhances HMGB3 expression via sponging miR-150-5p. (a) Transfection of miR-150-5p mimic increased miR-150-5p level, as determined by qRT-PCR assay. (b) miR-150-5p interacts with 3ʹ non-coding region of HMGB3. Bioinformatics analysis of binding site between LINC00857 and miR-150-5p using Starbase bioinformatic tool (left panel); WT or MUT luciferase reporter plasmids were transfected into SW480 and HCT116 cells for 48 h in the presence of miR-150-5p mimic or miR-NC. The luciferase activity was measured in each group (right panel). (c & d) NC inhibitor, miR-150-5p inhibitor, miR-NC, or miR-150-5p mimic was transfected into SW480 and HCT116 cells for 48 h. qRT-PCR and Western blot was used to determined HMGB3 expression level. (e & f) HMGB3 mRNA and protein expression was determined in cells of the following groups: sh-NC, sh-LINC00857, sh-LINC00857 + miR-150-5p inhibitor. qRT-PCR and Western blot was used to determined HMGB3 expression level. (g) HMGB3 expression in CRC tumor and non-carcinoma tissues was detected by PCR analysis (n = 50 pairs). (h) The expression levels of LINC0085*7* and HMGB3 in CRC tumors showed a significant positive correlation. (i) HMGB3 expression level was negatively correlated with miR-150-5p expression level in CRC tumors. *P < 0.05, **P < 0.01, ***P < 0.001

We next sought to identify potential target of miR-150-5p. Starbase predicts that there is a miR-150-5p binding site within the HMGB3 3ʹnon-coding region (Figure 6(b), left panel). We applied miR-150-5p mimic, which could increase miR-150-5p level (Figure 6(a)) to investigate whether miR-150-5p functionally interact with HMGB3 mRNA. Dual luciferase reporter assays showed that the transfection of miR-150-5p mimic significantly suppressed HMGB3-WT luciferase activity, but no inhibition was observed for HMGB3-MUT luciferase reporter (Figure 6(b), right panel). Consistently, miR-150-5p overexpression reduced HGMB3 mRNA and protein levels in HCT116 and SW480 cells, while miR-150-5p inhibitor increased HGMB3 mRNA and protein levels (Figure 6(c,d)). Furthermore, we investigated whether LINC00857 could act as a miRNA sponge that competes with miR-150-5p for HMGB3 mRNA. Silencing LINC00857 suppressed HMGB3 expression at mRNA and protein level, while miR-150-5p inhibitor rescued HMGB3 level after LINC00857 knockdown (Figure 6(e,f)).

We also examined HMGB3 mRNA level in CRC samples in relative to non-carcinoma samples. CRC tumor tissues showed a significantly higher level of HMGB3 mRNA (Figure 6(g)). Meanwhile, in CRC tumors samples, HMGB3 level was positively correlated with LINC00857 expression level, but was negatively correlated with miR-150-5p level (Figure 6(h,i)). Taken together, these findings indicate that LINC00857 promotes the expression of HMGB3 by competitively binding with miR-150-5P.

### Knockdown of LINC00857 inhibits CRC tumorigenesis in vivo

**Figure 7.**
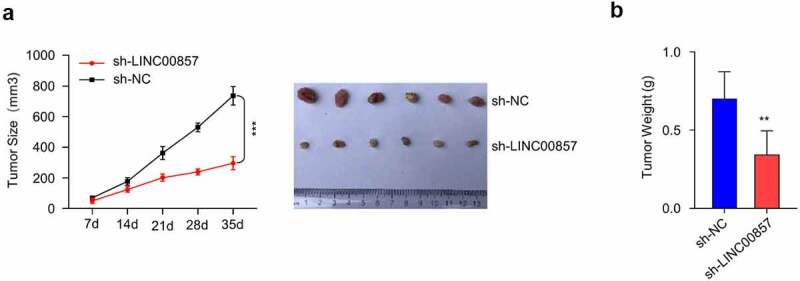
LINC00857 knockdown inhibits CRC tumorigenesis *in vivo.* HCT116 cells stably expressing sh-NC and sh-LIN00857 were injected into nude mice (n = 6 each), tumor size was monitored every 7 days. (a) sh-LIN00857 tumor growth was markedly inhibited as compared to sh-NC group. (b) The weight of tumors in sh-LIN00857 group was significantly lower as compared to sh-NC group. **P < 0.01, ***P < 0.001

In order to validate the oncogenic role of LINC00857 in vivo, nude mice were injected subcutaneously with HCT116 stably expressing sh-LINC00857 or sh-NC. We selected HCT116 cell since it expressed lowest level of LINC00857 and therefore we reasoned that the knockdown could bring the level close to that of normal cells (Figure 1(c)). We found that both the tumor weight and volume in sh-LINC00857 group were remarkably decreased as compared to sh-NC group, indicating that LINC00857 is necessary to support tumorigenesis of CRC cells *in vivo* (Figure 7(a,b)).

## Discussion

CRC remains as a major challenge for cancer treatment worldwide, which is the second largest contributor to cancer-associated mortality [33]. Increasing evidence indicates that lncRNAs are implicated in the molecular mechanism of the progression in various cancers [34,35]. Our work reveals the upregulation of LINC00857 in CRC samples and CRC cell lines, which seems to play an oncogenic role. Our results further suggest that the oncogenic role of LINC00857 is mediated by miR-150-5p/HMGB3 axis.

Several lncRNAs have been reported to contribute to carcinogenesis in multiple cancers [36]. For example, LINC00857 is upregulated in various cancer tissues, such as gastric cancer [37], ovarian cancer [38] and lung cancer [18]. Accordingly, we showed that LINC00857 expression is significantly increased in CRC cells and tissues. One previous study reported that the overexpression of LINC00857 could enhance the proliferation, invasion and colony formation of cancer cells [18]. Conforming to these findings, we showed that LINC00857 knockdown suppresses CRC cell proliferation, migration, invasion and induces cell apoptosis.

Previous studies have shown that miR-150-5p is frequently dysregulated in different cancer types [39–41]. Nonetheless, the regulation mechanisms by which miR-150-5p modulates the progression of CRC remain largely unknown. Considering that lncRNAs could regulate target gene by binding to specific miRNAs, we searched for the miRNA targets of LINC00857 and validated the functional interaction between miR-150-5p and LINC00857 in CRC cells. These data suggest that LINC00857/ miR-150-5p together regulate the malignant phenotype of CRC cells.

In addition, we identified that HMGB3, whose expression is positively correlated with LINC00857 in CRC cells, is a target of miR-150-5p. HMGB3 was reported to participate in cancer genesis and development, including gastric cancer [42] and breast cancer, leukemia [43]. Although a previous study demonstrated that HMGB3 could promote CRC proliferation and migration through modulating the WNT/β-catenin signal pathway [44], it remains unclear how HMGB3 is regulated in CRC cells. Our data showed that LINC00857 negatively interacts with miR-150-5p, which in turns maintains the expression of HMGB3 to support the malignant phenotype of CRC cells.

Increasing evidence suggests that abnormal lncRNAs expression is implicated in most of complex diseases. An increasing number of studies profiling lncRNAs are performed and produce massive genome-wide lncRNA data [6]. These datasets have been collected in various public databases [45–47]. Multiple computational models have been developed in order to identify disease-associated lncRNAs for further experimental validation [48–50]. Recently, Chang et. al. found that LINC00857 can promote CRC cell growth, migration, and invasion by sponging miR-1306 [51]. Together, our data and previous studies indicate that LINC00857 may have multiple targets in regulating CRC progression.

Although our study demonstrated the relationship between LINC00857 and clinicopathological features of CRC, the present work has some limitations. The mechanism of LINC00857 upregulation remains unclear; the functional role of miR-150-5p in controlling CRC tumorigenesis need to validated in animal model.

## Conclusion

Our study provided evidence that LINC00857 expression is significantly increased in CRC cells and tissues. LINC00857 overexpression is required for CRC cell proliferation, migration and invasion. LINC00857 upregulation maintains HMGB3 protein expression by sponging miR-150-5p. In addition, LINC00857 knockdown remarkably suppresses CRC tumorigenesis in mouse xenograft model. These data suggest that LINC00857 could serve as a novel therapeutic target for CRC treatment.

## Data Availability

The data is available from the corresponding author upon reasonable request.

## Highlights

- LINC00857 is upregulated in CRC tissues and cell lines;
- LINC00857 upregulation is required for the malignant features of CRC cells;
- LINC00857 maintains HMGB3 expression by sponging HMGB3;
- Silencing LINC00857 suppresses CRC tumorigenesis *in vivo*.
